# Prevalence of Postpartum Depression Among Women Attending Primary Care in Southern Mexico: A Cross-Sectional Study

**DOI:** 10.7759/cureus.106234

**Published:** 2026-03-31

**Authors:** Aziz Curioca-Velásquez, Gloria Y Santos-Herrera, Alfredo González-Ayala, Nallely Rincón Peregrino, Quitzia L Torres-Salazar

**Affiliations:** 1 Family Medicine, Hospital General de Zona No. 2 con Medicina Familiar, Salina Cruz, Oaxaca, MEX; 2 Biomedical Sciences, Universidad Juárez del Estado de Durango, Durango, MEX

**Keywords:** edinburgh postnatal depression scale, maternal mental health, postpartum depression, primary care, public health

## Abstract

Background and objective

Postpartum depression (PPD) is a frequent maternal mental health disorder associated with adverse outcomes for both mothers and their children. Its burden may be particularly relevant in regions characterized by social vulnerability and limited access to specialized mental health services. Epidemiological data from southern Mexico remain scarce, especially in primary care settings where early identification is critical. The objective of this study is to estimate the prevalence of PPD among women attending a primary care unit in Salina Cruz, Oaxaca, Mexico.

Methods

A cross-sectional study was conducted among postpartum women attending a family medicine unit of the Instituto Mexicano del Seguro Social in Salina Cruz, Oaxaca. Postpartum depressive symptoms were assessed using the Edinburgh Postnatal Depression Scale. Sociodemographic and obstetric variables were recorded. Prevalence was estimated with 95% CIs. Differences between women with and without probable PPD were explored using the chi-square or Fisher’s exact test, as appropriate.

Results

A total of 120 postpartum women were included. The median age was 25.5 years (q25-q75: 20-32), and the median time from delivery to screening was six weeks (q25-q75: 4-6). The median EPDS score was 11 (q25-q75: 10-12). Using a cutoff score of ≥13, the prevalence of PPD was 21.7% (95% CI: 14.3-29.1). Cesarean section accounted for 65.0% of deliveries, and breastfeeding difficulties were reported by 59.2% of participants.

Conclusions

PPD was identified in approximately one in five women attending primary care in southern Mexico. In a referral setting serving a socioeconomically diverse population with limited access to specialized mental health services, this prevalence represents a clinically relevant and potentially underrecognized burden. Routine screening for PPD in primary care could facilitate earlier detection and improve access to timely mental health care.

## Introduction

Postpartum depression (PPD) is one of the most common complications of the puerperium and represents a significant public health concern worldwide. Global estimates suggest that approximately 10-20% of women experience clinically relevant depressive symptoms during the first year after childbirth, although prevalence varies widely depending on geographic region, screening tools, and sociocultural context. In low- and middle-income countries, reported rates are often higher, reflecting disparities in access to healthcare, social support systems, and mental health services [1].

PPD is associated with substantial maternal and neonatal consequences. Affected women may experience impaired mother-infant bonding, difficulties in breastfeeding, decreased adherence to pediatric follow-up, and reduced functional capacity. Long-term consequences include persistent depressive disorders and adverse neurodevelopmental outcomes in children [2]. In Mexico, epidemiological data remain variable across regions and healthcare settings. A study conducted in postpartum Mexican women reported a prevalence of depressive symptoms of 39.2% (95% CI: 34-45%) using the Edinburgh Postnatal Depression Scale (EPDS) [3], substantially higher than global pre-pandemic estimates ranging from 6.9% to 20% in high-income settings. Although contextual factors may influence these estimates, the marked variability observed nationwide highlights important territorial gaps and underscores the need for region-specific data, particularly in areas where systematic screening is not routinely implemented and mental health resources are limited [4].

Within this national context, southern Mexico represents a region of particular epidemiological relevance. The state of Oaxaca has been consistently ranked among the states with the highest levels of social vulnerability in the country. Approximately 65.9% of its population lives in poverty, life expectancy remains below the national average, and substantial proportions of households lack adequate water sources or sanitary services. In addition, disparities in educational attainment and healthcare access persist across its municipalities [5]. In this setting, primary care units of the Instituto Mexicano del Seguro Social (IMSS) frequently function as first contact and referral centers for both urban and rural populations, positioning them as strategic sites for the early detection of maternal mental health disorders [6].

Despite this, systematic screening for PPD is not routinely implemented in many primary care settings, and the availability of specialized mental health services remains limited. In this context, the objective of this study was to estimate the prevalence of PPD among women attending a primary care unit in Salina Cruz, Oaxaca, Mexico.

## Materials and methods

A cross-sectional prevalence study was conducted between January and June 2025 at a family medicine unit of the IMSS located in Salina Cruz, Oaxaca, Mexico. This primary care unit functions as a first contact and referral center for both urban and surrounding rural populations in the Isthmus region of southern Mexico. The study was designed and reported in accordance with the STrengthening the Reporting of OBservational studies in Epidemiology (STROBE) statement and the recommendations of the EQUATOR Network for observational prevalence research.

Women in the puerperium, defined as up to 12 months postpartum, who attended the outpatient primary care clinic during the study period were invited to participate through consecutive sampling to reduce selection bias. Eligible participants were women aged 18 years or older, within 12 months after delivery, attending routine follow-up consultations, and willing to provide written informed consent. Women with a previous diagnosis of major depressive disorder prior to pregnancy, those receiving current psychiatric treatment at the time of evaluation, and those unable to complete the questionnaire due to cognitive or language limitations were excluded to ensure accurate identification of probable postpartum depressive symptomatology.

The sample size was calculated for prevalence estimation using a 95% confidence level, an expected prevalence of 20% based on prior national estimates, and a margin of error of 7%, yielding a minimum required sample of 120 participants.

Data were collected through structured face-to-face interviews conducted in a private clinical setting to ensure confidentiality. Sociodemographic variables included age, educational level, marital status, employment status, and place of residence (urban or rural). Obstetric variables included parity, mode of delivery, and history of obstetric complications. When necessary, medical records were reviewed to corroborate clinical information.

Postpartum depressive symptoms were assessed using the EPDS [3], a validated 10-item self-administered screening instrument widely used in postpartum populations. A cutoff score of ≥13 was used to define probable PPD, consistent with validation studies in Spanish-speaking populations.

Descriptive statistics were used to summarize participant characteristics. Continuous variables were reported as mean ± SD or median with IQR, depending on distribution. Categorical variables were presented as frequencies and percentages. The prevalence of PPD was calculated with 95% CIs. Differences between women with and without probable PPD were explored using the chi-square test or Fisher’s exact test, as appropriate. A two-sided p-value <0.05 was considered statistically significant. Statistical analyses were performed using IBM SPSS Statistics for Windows, Version 27.0 (Released 2019; IBM Corp., Armonk, NY, USA).

The study was conducted in accordance with the principles of the 2024 update of the Declaration of Helsinki and the Mexican General Health Law on Health Research. The research protocol was reviewed and approved by the Institutional Research Committee of the IMSS (approval R-2024-2001-048). All participants provided written informed consent prior to inclusion, and confidentiality and data anonymization were strictly maintained throughout the study.

## Results

A total of 120 postpartum women were included in the analysis. The median age was 25.5 years (q25-q75: 20-32), and the median time from delivery to screening was six weeks (q25-q75: 4-6). Cesarean section accounted for 65.0% of births. Breastfeeding difficulties were reported by 71 women (59.2%).

The median EPDS score in the overall population was 11 (q25-q75: 10-12). Using the predefined cutoff score of ≥13, 26 women screened positive for PPD, corresponding to a prevalence of 21.7% (95% CI: 14.3-29.1) (Table 1).

**Table 1:** Baseline characteristics of the study population (N = 120) Prevalence: 21.7% (95% CI: 14.3-29.1) EPDS, Edinburgh Postnatal Depression Scale; PPD, postpartum depression

Variable	Overall (N = 120)
Age, years, median (q25-q75)	25.5 (20-32)
Weeks postpartum at screening, median (q25-q75)	6 (4-6)
Cesarean delivery, n (%)	78 (65.0%)
Vaginal delivery, n (%)	42 (35.0%)
Breastfeeding difficulties, n (%)	71 (59.2%)
EPDS score, median (q25-q75)	11 (10-12)
PPD (EPDS ≥13), n (%)	26 (21.7%)

When stratified by EPDS result, the median age was 23.5 years (q25-q75: 19-30) in women with PPD and 26.5 years (q25-q75: 20-32) in women without PPD (p = 0.444). The median time since delivery was six weeks in both groups (p = 0.570). Cesarean delivery occurred in 69.2% of women with PPD and 63.8% of those without PPD (p = 0.609). Breastfeeding difficulties were reported by 65.4% of women with PPD and 57.4% of women without PPD (p = 0.507). No statistically significant differences were identified between groups (Table 2).

**Table 2:** Sociodemographic and obstetric characteristics of postpartum women, stratified by PPD screening status ^*^ Differences analyzed using the Mann-Whitney U test ^**^ Differences analyzed using the chi-square test PPD, postpartum depression

Variable	PPD (n = 26)	No PPD (n = 94)	Test value	p-Value
Age, years, median (q25-q75)^*^	23.5 (19-30)	26.5 (20-32)	1342	0.444
Weeks postpartum, median (q25-q75)^*^	6 (4-8)	6 (4-6)	1141	0.57
Cesarean delivery, n (%)^**^	18 (69.2%)	60 (63.8%)	0.261	0.609
Vaginal delivery, n (%)^**^	8 (30.8%)	34 (36.2%)	0.261	0.609
Breastfeeding difficulties, n (%)^**^	17 (65.4%)	54 (57.4%)	0.531	0.507
Weeks of pregnancy, median (q25-q75)^*^	39 (38-39)	38 (38-40)	1259.5	0.804
Surviving children, median (q25-q75)^*^	2 (1-2)	2 (1-2)	983.5	0.095

## Discussion

In this cross-sectional study conducted in a primary care unit in southern Mexico, PPD was identified in 21.7% (95% CI: 14.3-29.1) of women during the first year after childbirth. The median EPDS score in the overall population was 11 (q25-q75: 10-12), indicating that a substantial proportion of women were clustered near the clinical cutoff threshold. This finding suggests a clinically relevant burden of depressive symptomatology even among those not meeting criteria for probable PPD.

The observed prevalence of 21.7% is slightly above the upper limit of global estimates reported in high-income settings, which range between 10% and 20% [7], and is consistent with pooled international estimates described in meta-analytic data [8]. Large meta-analyses evaluating perinatal depressive symptoms across diverse populations have reported considerable global heterogeneity, with pooled prevalence estimates frequently ranging between 17% and 25%, depending on geographic region, screening tools, and socioeconomic context. These studies consistently show higher prevalence rates in low- and middle-income countries, where structural determinants such as poverty, limited access to mental health services, and social stressors may amplify maternal psychological vulnerability [9]. However, it remains lower than the 39.2% prevalence reported in Mexican postpartum women during the COVID-19 lockdown [4]. This difference may reflect contextual factors, including pandemic-related stressors, social isolation, and economic instability that disproportionately affected maternal mental health during that period. Nevertheless, the persistence of a prevalence exceeding 20% in a nonpandemic context underscores that postpartum depressive symptomatology remains a substantial public health concern in Mexican women.

Importantly, this study was conducted in Salina Cruz, Oaxaca, a state characterized by high levels of social vulnerability. Oaxaca has consistently ranked among the Mexican states with the highest poverty rates, with approximately 65.9% of its population living in poverty, reduced life expectancy compared to the national average, and significant deficits in access to water, sanitation, and educational attainment [5]. These structural determinants may influence maternal mental health through chronic stress exposure, economic insecurity, and limited access to specialized psychological care. Qualitative research conducted among Latina mothers has also highlighted the role of cultural expectations and community beliefs in shaping the experience and expression of PPD. In focus group studies, many Latina mothers report feeling pressure to suppress emotional distress to maintain their perceived role as the emotional and functional core of the family, often fearing judgment or social stigma if depressive symptoms are openly expressed [10]. Such cultural dynamics may contribute to the normalization or concealment of depressive symptoms, further complicating early recognition and help-seeking behaviors among mothers in socially vulnerable settings. In this context, primary care units of the IMSS often serve as first-contact and referral centers for both urban and rural populations, making them critical sites for early detection of PPD [6].

Despite the elevated prevalence, no statistically significant differences were identified between women with and without probable PPD across sociodemographic or obstetric variables. The absence of detectable differences may reflect limited statistical power to identify small effect sizes or may suggest that postpartum depressive symptomatology in this setting is not confined to specific demographic subgroups but rather distributed across the population. This observation aligns with the notion that structural and contextual stressors operate broadly at the community level rather than exclusively affecting predefined high-risk profiles [11].

Similarly, van der Zee-van den Berg et al. reported that although several individual risk factors for PPD may be identified, associations with sociodemographic characteristics are often inconsistent across community-based populations. In their large community cohort of 1,406 mothers, factors such as a history of depression, low maternal self-efficacy, and poor maternal health were more strongly associated with depressive symptoms than most demographic variables. The authors also highlight that demographic characteristics frequently show heterogeneous or weak associations with PPD across studies, reinforcing the complexity of identifying consistent risk profiles in population-based samples [12].

Breastfeeding difficulties were reported by 65.4% of women with PPD and 57.4% of women without PPD, although this difference did not reach statistical significance (p = 0.507). While causality cannot be inferred from this cross-sectional design, the high overall frequency of reported lactation difficulties (59.2%) merits attention. Previous literature has documented bidirectional relationships between depressive symptoms and breastfeeding challenges, wherein depressive symptomatology may impair maternal confidence and bonding, while breastfeeding difficulties may exacerbate psychological distress [8]. This relationship has been further supported by recent evidence synthesized in a systematic review and meta-analysis by Ahmadinezhad et al. [13], which demonstrated that PPD is significantly associated with lower breastfeeding self-efficacy scores compared with nondepressed mothers. Their pooled analysis showed statistically significant reductions in maternal breastfeeding self-efficacy during the early postpartum period, including the first and third days after delivery, suggesting that depressive symptomatology may directly undermine maternal confidence in breastfeeding and contribute to early lactation difficulties. In resource-constrained settings, insufficient lactation support services could further compound this dynamic.

The median EPDS score of 11 (q25-q75: 10-12) is particularly noteworthy, as it indicates that half of the study population scored within two points of the diagnostic threshold. This clustering near the cutoff suggests that a proportion of women may fluctuate around clinically relevant symptom levels, potentially remaining undiagnosed in the absence of systematic screening. Given that PPD is not routinely screened in many primary care settings in Mexico, including parts of southern regions, the potential for missed cases is substantial.

From a public health perspective, the identification of probable PPD in approximately one in five women attending a primary care unit in a socially vulnerable region reinforces the need for routine screening strategies. The EPDS is brief, low-cost, and easily implementable in first-level care settings. Integrating structured mental health screening into postpartum follow-up visits could facilitate earlier identification and referral, particularly in regions where access to psychiatric services is limited [14]. This need is further supported by the work of Place et al., who report that PPD may affect up to one quarter of women during the first year after childbirth but remains one of the most underdiagnosed pregnancy-related health conditions due to multiple barriers to help-seeking and access to care. Their analysis highlights that barriers occur at multiple levels (including individual stigma, limited provider engagement, and structural healthcare access limitations), underscoring the importance of systematic screening strategies within primary care settings to improve detection and timely intervention [15].

This study has several strengths. It provides region-specific data from southern Mexico, an area underrepresented in national epidemiological reports. The use of a validated screening instrument and the implementation of consecutive sampling strengthen internal validity. However, certain limitations should be acknowledged. The cross-sectional design precludes causal inference. The EPDS is a screening tool rather than a diagnostic instrument, and clinical confirmation was not performed. Additionally, the sample size, while adequate for prevalence estimation, may limit the ability to detect subtle subgroup differences.

## Conclusions

PPD was identified in approximately one in five women attending primary care in southern Mexico, indicating a substantial burden of depressive symptoms during the postpartum period. In a region characterized by pronounced social vulnerability and limited availability of specialized mental health services, this prevalence underscores the potential for underdiagnosis when systematic screening is not routinely implemented. These findings highlight the critical role of primary care as a strategic point for the early identification of maternal mental health disorders. Implementing structured screening strategies during postpartum follow-up visits may facilitate timely detection, appropriate referral, and improved maternal mental health outcomes.
